# Xanthoma Disseminatum with Tumor-Like Lesion on Face

**DOI:** 10.1155/2014/621798

**Published:** 2014-04-10

**Authors:** Habib Ansarin, Hoda Berenji Ardestani, Seyed Mehdi Tabaie, Nasrin Shayanfar

**Affiliations:** ^1^Department of Dermatology, Hazrat-e Rasool University Hospital, Iran University of Medical Sciences, Tehran, Iran; ^2^Skin and Stem Cell Research Center, Tehran University of Medical Sciences, Kamraniye Street, No. 4, Maryam Alley, Tehran 1937957511, Iran; ^3^Iranian Center for Medical Laser, Academic Center for Education, Culture and Research, Tehran, Iran; ^4^Department of Pathology, Iran University of Medical Sciences, Tehran, Iran

## Abstract

Xanthoma disseminatum (XD) is a rare benign mucocutaneous xanthomatosis that is classified as a benign non-Langerhans cell histiocytosis. We report a 62-year-old man who presented with widespread yellow-brown papulonodular and tumoral lesions on face, flexors, and trunk. Histopathological features of the cutaneous lesions were typical of XD.

## 1. Introduction

Xanthoma disseminatum (XD) is a rare benign mucocutaneous xanthomatosis classified as a benign form of non-Langerhans cell histiocytosis [1–3]. Prominent flexural xanthomatous lesions and a frequent association with diabetes insipidus are characteristics of the disease. There is a high rate of mucosal lesions, as well as meningeal involvement leading to diabetes insipidus, but other viscera are rarely involved [3, 4]. We report a case of XD which had widespread yellow-brown papulonodular lesions on face, flexors, and trunk.

## 2. Case Report

**Figure 1:**
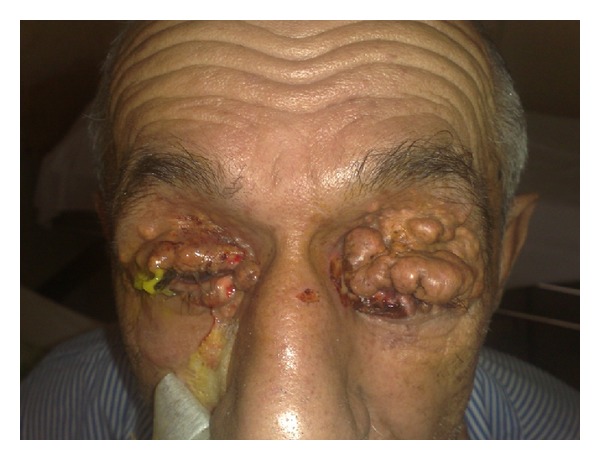
Tumor-like lesions on periorbital area, the ulceration like lesion do to biopsy's site.

**Figure 2:**
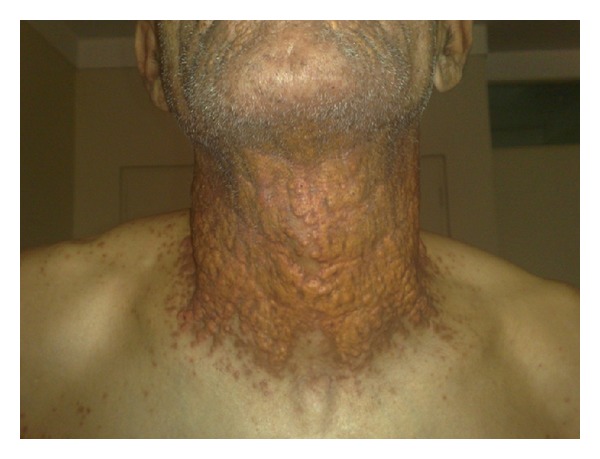
Involvement of flexural regions of neck with papule that confluent to plaque lesion.

A 62-year-old man was admitted to our department due to a gradually evolving disseminated papulonodular eruption on face, flexors, and trunk for 30 years ago. On examination, multiple, well-defined, yellowish brown papules and nodules and tumor-like lesions were seen symmetrically on upper and lower eyelids (Figure 1). Such papules also were observed on the cheeks and perioral region. On the anterior of the neck the lesions were confluent and formed a diffused plaque (Figure 2).

**Figure 3:**
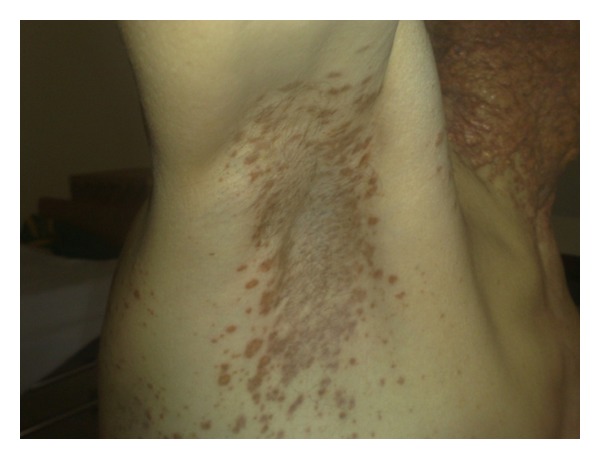
Involvement of red-brown papules.

**Figure 4:**
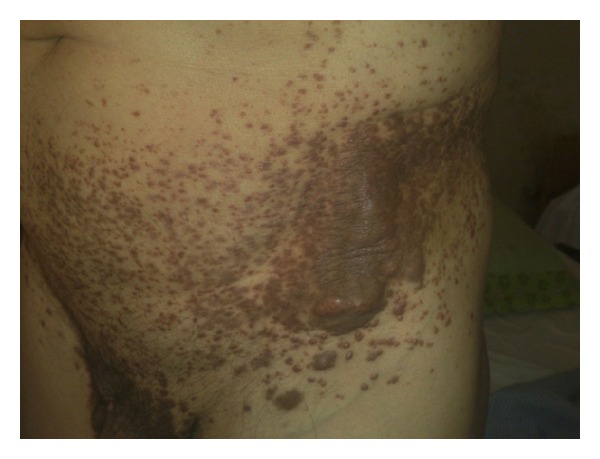
Papules, plaque, and tumor like lesions.

**Figure 5:**
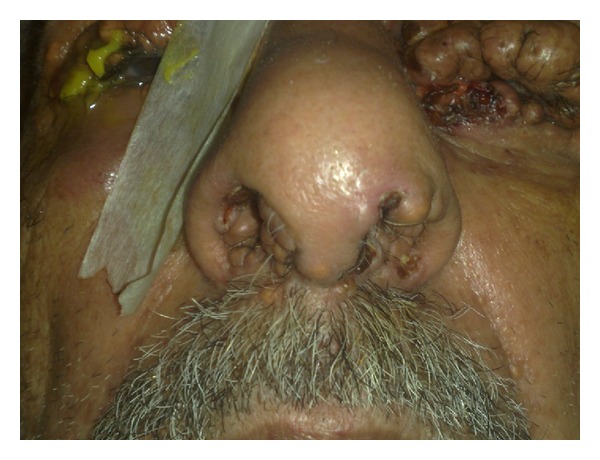
Involvement of nasal mucosa.

Hundreds of red to brown papules were distributed symmetrically on axilla, genitalia, poplitea, and trunk, some of which are confluent together and formed plaque or tumor-like lesions (Figures 3 and 4). There were also some papule and nodules on nasal mucosa (Figure 5).

He had no history of polyuria or any previous medical history.

Analysis at that point did not reveal any abnormalities in urine osmolality. Laboratory findings revealed a white blood cell count of 10,300 cells/mL containing 74% neutrophils. C-reactive protein was 7 mg/dL (normal values of 0.5 mg/dL).

Fasting cholesterol was 148 (normal values up to 200) and triglyceride was 141 (normal values up to 150).

Chest X-ray was normal.

Magnetic resonance imaging of the brain and pituitary revealed no abnormalities.

**Figure 6:**
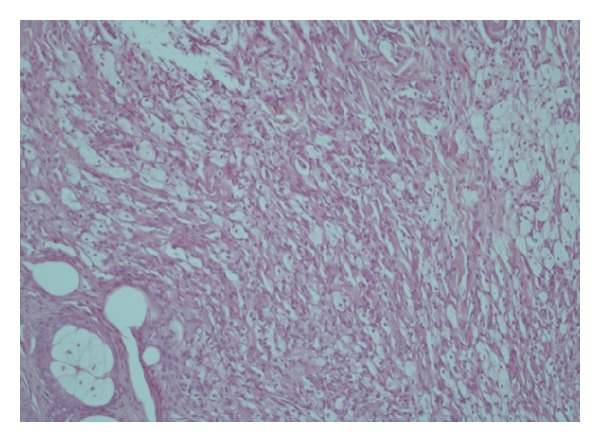
Dense dermal diffuse histiocytic infiltration.

**Figure 7:**
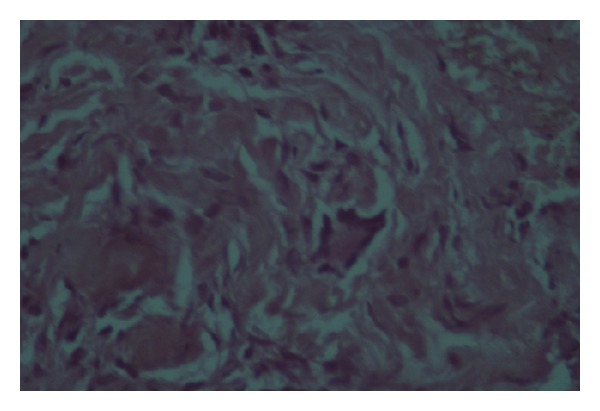
A dense dermal diffuse histiocytic infiltration interspersed with mixed inflammatory cells and giant cells.

**Figure 8:**
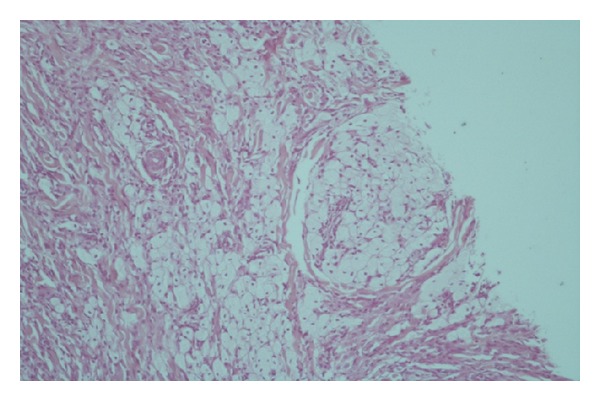
Foam cells were also seen.

**Figure 9:**
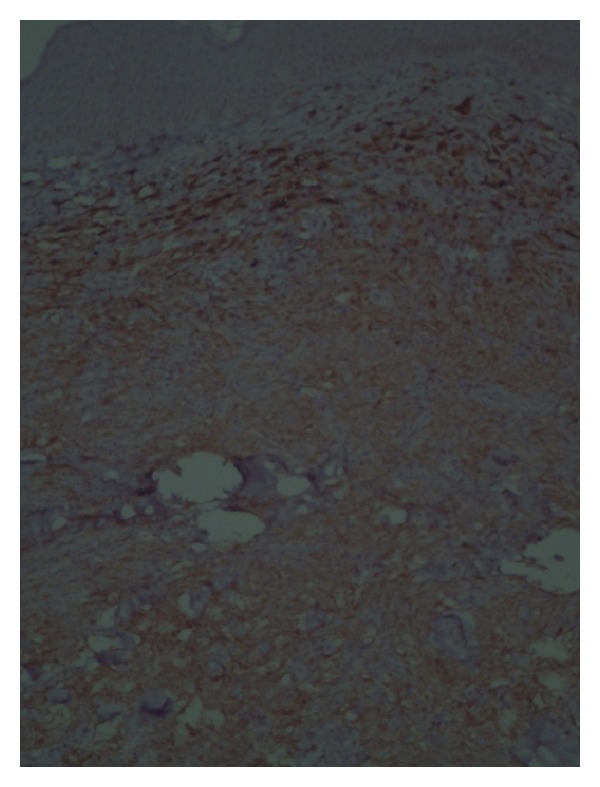
Immunohistochemistry was positive.

Histopathologic examination of skin biopsies showed a dense dermal diffuse histiocytic infiltration interspersed with mixed inflammatory cells and giant cells (Figures 6 and 7). Abundant foam cells were also seen (Figure 8). A neural infiltration was not present. Immunohistochemistry was positive for CD68 (KP1) (Figure 9) and negative for S-100 protein.

## 3. Discussion

Xanthoma disseminatum (XD) is a rare but distinct sporadic disorder, in which lipid deposition occurs secondary to a proliferation of histiocytic cells. This is usually seen before 25 years, as rarely reported in the elderly [1]. XD is characterized by numerous features like widely disseminated but often closely set and even coalescing, round to oval, orange or yellow-brown papules, and nodules found mainly on the flexor surfaces, such as neck, axillae, antecubital fossae, groin, and perianal region. Often there are lesions around the eyes. The mucous membranes are affected in 40% to 60% of cases. In addition to oral lesions, there may be pharyngeal and laryngeal involvement.

The etiology of XD is unknown. It has been suggested that XD represents a reactive proliferation of histiocytes with secondary accumulation of lipid. But it is not associated with hyperlipidemia.

Three patterns have been identified; the most common pattern is the persistent form. Rarely, lesions may regress spontaneously, and even more infrequently in the progressive form there may be significant internal organ involvement [2]. XD consists of the triad of widespread normolipidemic xanthomata, mucous membrane involvement of the upper respiratory tract, and mild transient diabetes insipidus [1].

Mucous membrane involvements of XD have been reported in 40–60% of cases [4]. The most frequently affected mucosal areas are larynx, pharynx, mouth, trachea, and conjunctiva, although in postmortem studies involvement of the esophagus and stomach was reported as well [5]. Our patient had multiple xanthomatous papules and nodules in his nasal mucosa, but investigation for involvement of other mucosal regions was not carried out.

Meningeal involvement is common, leading to diabetes insipidus when infiltration at the base of the brain is present. This condition is encountered in about 40% of cases but usually is less severe than that associated with Langerhans cell disease. Characteristically, internal lesions other than diabetes insipidus are absent. Our patient did not have any sign or symptom of diabetes insipidus [6].

In a few instances multiple osteolytic lesions have been found, especially in the long bones, as well as lung and central nervous system infiltrates.

Histopathologically, in early lesions, scalloped macrophages dominate the histologic picture, with few foamy cells. Well-developed lesions may still show scalloped cells, but xanthomatization occurs in most cases. Most well-developed lesions contain a mixture of scalloped cells, foamy cells, and inflammatory cells, as well as Touton and foreign-body giant cells. XD histiocytes stain for lysozyme and aI-antitrypsin and also express CD68, CDllb, CD14, CDllc, and factor XIIIa [7].

The main differential diagnosis of XD is generalized eruptive histiocytosis (GEH) and progressive nodular histiocytosis (PNH). Multiple skin lesions occurring in adolescence or young adulthood with prominent involvement of flexural areas, as well as viscera and mucosa, and comprising mainly xanthomatous cells are XD; multiple lesions appearing in crops, generally sparing the flexures, and occurring in normolipemic patients are GEH, while multiple lesions arising in skin of an older patient and progressing to form large nodules, with no evidence of spontaneous regression and comprising mainly spindle-shaped histiocytes, are PNH [8].

We report this case for its unusual large tumor-like lesions around the eyes. In the literature, some authors have previously described cases of XD with eyelid or periocular accentuation of lesions [9, 10].

There are various treatment modalities, like vasopressin, corticosteroids, antiblastic chemotherapy, radiotherapy, cryotherapy, CO_2_ LASER therapy, and surgical resection, used alone or in combination [3, 8]. Oral prednisolone (2 mg/kg/day) and azathioprine (2 mg/kg/day) did not show significant efficacy; a combination of lipid-lowering agents or azathioprine and cyclophosphamide was reportedly useful [3]; combination of systemic steroids, clofibrate, and chemotherapy was effective in some studies [3]. Bone marrow transplantation has been used successfully in life-threatening XD [11]. 2-Chlorodeoxyadenosine therapy was found useful in maintaining remission and long-term control of cutaneous lesions [12].
